# Evaluation of the efficacy and safety of hydrogen peroxide in the treatment of molluscum contagiosum in children compared to potassium hydroxide: a pilot study^☆^

**DOI:** 10.1016/j.abd.2023.10.007

**Published:** 2024-08-05

**Authors:** Elinah Narumi Inoue, Felipe de Paula Saboia, Amanda Hertz, Marcia Olandoski, Dâmia Kuster Kaminski Arida

**Affiliations:** a Department of Dermatology, Hospital Universitário Evangélico Mackenzie, Curitiba, PR, Brazil; b Department of Statistics, Pontifícia Universidade Católica do Paraná, Curitiba, PR, Brazil

Dear Editor,

Molluscum contagiosum (MC), an infectious dermatosis caused by a virus of the *Poxviridae* family,^1^ mainly affects children, with transmission occurring through direct contact.^2^ It presents as 2 to 3 mm, rounded, pink, or normochromic papules, with central umbilication and a plug of caseous material.^3^

Although considered a self-limited disease, its treatment alleviates discomfort, and prevents infectious complications, transmission, and self-inoculation,^3^ refraining social marginalization of the patient and parental anxiety.

There are several types of therapy for MC^2^, ^3^, ^4^ but many of these methods can generate uncomfortable effects that make adherence difficult, leading to therapeutic failure, anxiety and psychological trauma for children and families.^4^ A widely studied method is a 5% potassium hydroxide (KOH) solution, which, despite being effective, causes uncomfortable side effects, such as pain and dyschromia.^5^ Therefore, it is necessary to search for other treatment options that may bring more comfort and adherence to therapy. A promising option is hydrogen peroxide (H_2_O_2_). It has antimicrobial action through the oxidation of viral molecules, damaging their DNA and leading to cytotoxicity,^6^ but without major damage to adjacent tissue. Thus, its adverse effects are generally mild.^7^ However, there is still a scarcity of studies demonstrating its real efficacy and safety.^8^

Accordingly, this study evaluated the efficacy and safety of using H_2_O_2_ 1% cream as MC treatment in pediatric patients. And, it was comparared with KOH 5% solution, through a double-blind, randomized, placebo-controlled pilot study with 30 patients with MC, aged 2 to 16 years, who had had no treatment for the disease in the previous six months, randomly allocated into three treatment groups following the order of arrival. Group A was submitted to treatment with H_2_O_2_ 1% cream; Group B, treatment with KOH 5% solution; Group C, treatment with Lanette cream (placebo), all applied twice a day, continuously used until the lesions became irritated. The participants were evaluated every 4 weeks for three months through photographic records and clinical evaluation by a dermatologist blinded to the intervention, regarding the number of lesions, their reduction, and side effects. Parents perception was also recorded throughout a specific questionnaire. Of the 30 patients, seven did not complete the study (five due to poor adherence to treatment; one due to loss of follow-up; and one due to an adverse effect of KOH), leaving 23 individuals - eight in Group A, seven in Group B and eight in Group C.

Regarding the percentage reduction of lesions (Fig. 1), in the 4th week, there was a greater reduction of 50% of lesions in almost 40% of the patients in Group A, close to Group B (42.9%; p = 1) and higher than Group C (25%; p = 1). In the 12th week, this reduction reached 85% of the patients in Group A, lower than in Group B (100%), but higher than in Group C (62.5%; p = 1).

**Figure 1:**
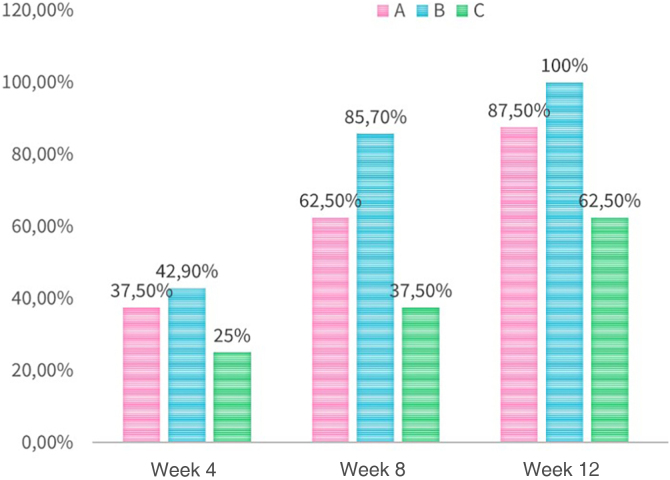
Reduction of ≥ 50% of lesions (A, Hydrogen peroxide; B, Potassium hydroxide; C, Placebo).

The absolute reduction in the number of total lesions (Fig. 2), considering the sum of all patients, was 20.7% in Group A in the 4th week, lower than that obtained in Group B (41.2%; p = 0.265). Both Group A and Group B showed higher values than the placebo group, which on the other hand showed an increase in the number of lesions during this period. In the 12th week, 73.2% of the lesions had resolved in Group A (p = 0.034), lower than the 98.4% reduction seen in Group B (p = 0.034), but higher than in Group C (45.5%; p = 0.034).

**Figure 2:**
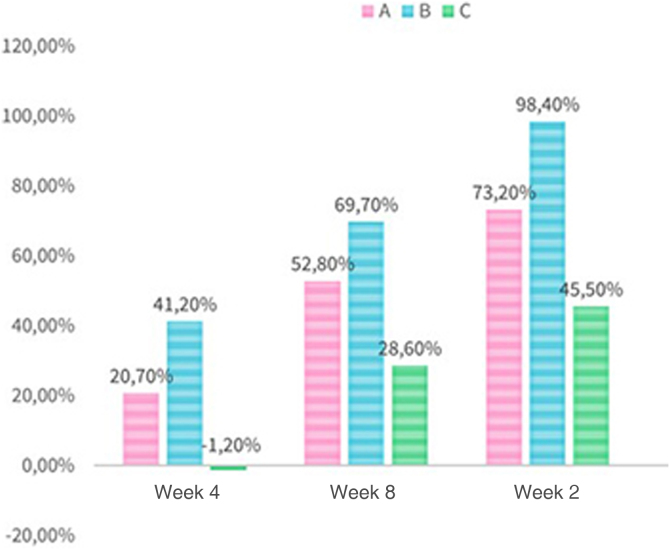
Absolute reduction of total lesions (A, Hydrogen peroxide; B, Potassium hydroxide; C, Placebo).

It was also observed that Group A showed greater variability in results between patients, while Group B showed almost no dispersion. Although Group C also had cases of improvement, there were cases of worsening of the condition (Fig. 3).

**Figure 3:**
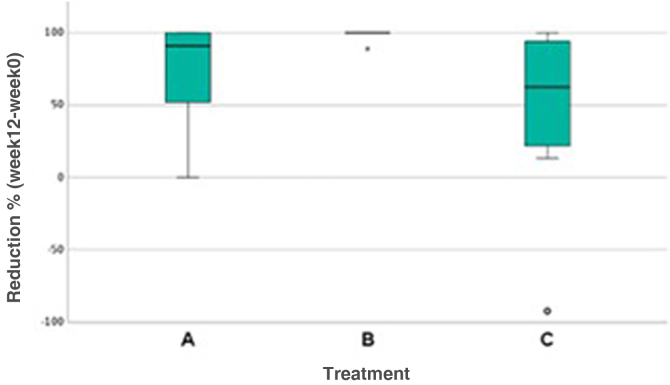
Analysis of the number of lesions (A, Hydrogen peroxide; B, Potassium hydroxide; C, Placebo).

Regarding parents perception of side effects, from the 4th to 12th week (p = 1, p = 1, p = 0.846), 50% of the patients in Group A experienced some adverse event. In Group B, 70% of the patients experienced adverse effects in the 4th week, 42.9% in the 8th week and more than 80% in the 12th week (p = 1, p = 1, p = 0.846, respectively). There were also reports of adverse effects in Group C, 37.5%, 25% and 75% in weeks 4, 8 and 12, respectively, which can be attributed to symptoms of the natural evolution of the disease.^3^

Of the reported side effects, erythema was present in 37.5% of the patients in Group A in the 4th week, similar to what was found in Group C (37.5%; p = 1) and lower than that in Group B (57.1%; p = 1). In the 12th week, Group A showed more erythema when compared to the other groups (A: 37.5%; B: 14.3%; C: 12.5%; p = 1).

As for crusts, they were present in 25% of the patients in Group A in the 4th week and absent in the 8th and 12th weeks, while in Group B, they were present in 14.4% throughout all weeks, close to the values in Group C (13%; p = 1).

As for erosions/ulcers, Groups A and C did not show them in any of the evaluations, whereas 14.3% of all patients in Group B had these signals in the 12th week.

Pruritus was more prevalent in Group A compared to the two other groups in all weeks, affecting 25% of children in the 12th week. Group B, on the other hand, did not report pruritus, unlike other studies, in which this symptom is a common finding.^8^

Burning/pain sensation was present in 12.5% of individuals in Group A in all weeks, the same as in Group C (12.5%). In Group B, this value was almost four times higher in the 4th week (57.1%; p = 0.357); and reached 71.4% of the patients in the 12th week (p = 0.122).

In Group A, 25% of the children had dyschromia in the 12th week, lower than what was seen in Group C (37.5%, p = 1) and also Group B, which had 71.4% in the 4th week (p = 0.021), with persistence of the condition in almost half of the patients in the 12th week (42.9%; p = 1).

It can be concluded that the use of H_2_O_2_ showed a tendency towards superior efficacy in relation to the placebo, but still lower than that of KOH. However, it seems to be a promising therapy due to its safety and lower incidence of side effects such as burning sensation, pain and dyschromia. Due to these findings, it may be a good option for younger children, with more sensitive skin and intolerant to the adverse manifestations caused by KOH. As this is a pilot study with a small sample, it did not obtain the necessary statistical significance to allow a conclusion on the real effectiveness of H_2_O_2_, requiring further larger sample studies to guarantee statistically significant efficacy and safety results.

## Financial support

We had financial support from FUNADERM (Fundo de Apoio à Dermatologia).

## Authors’ contributions

Elinah Narumi Inoue: Data collection, analysis and interpretation of data; drafting and editing of the manuscript; collection, analysis and interpretation of data; critical review of the literature.

Felipe de Paula Saboia: Data collection.

Amanda Hertz: Data collection.

Marcia Olandoski: Statistical analysis.

Dâmia Kuster Kaminski Arida: Design and planning of the study; data collection, or analysis and interpretation of data; drafting and editing of the manuscript or critical review of important intellectual content; collection, analysis and interpretation of data; effective participation in research orientation; intellectual participation in the propaedeutic and/or therapeutic conduct of the studied cases; approval of the final version of the manuscript.

## Conflicts of interest

None declared.

## References

[1] Rodríguez G., Arenas D. (2019). Molusco contagioso. Rev Asoc Colomb Dermatol Cir Dermatol.

[2] Leung A.K.C., Barankin B., Hon K.L.E. (2017). Molluscum contagiosum: un update. Recent Pat Inflamm Allergy Drug Discov.

[3] Meza-Romero R., Navarrete-Dechent C., Downey C. (2019). Molluscum Contagiosum: an update and review of new perspectives in etiology, diagnosis, and treatment. Clin Cosmet Investig Dermatol.

[4] Forbat E., Al-Niaimi F., Ali F.R. (2017). Molluscum Contagiosum: review and update on management. Pediatr Dermatol.

[5] Rajouria E.A., Amatya A., Karn D. (2011). Comparative study of 5% potassium hydroxide solution versus 0.05% tretinoin cream for Molluscum Contagiosum in children. Kathmandu Univ Med J (KUMJ).

[6] Mcdonnell G., Rappoport Z. (2014). PATAI’S Chemistry of Functional Groups.

[7] Schianchi R., Nazzaro G., Veraldi S. (2018). Treatment of molluscum contagiosum with hydrogen peroxide. Clin Exp Dermatol.

[8] Van der Wouden J.C., van der Sande R., Kruithof E.J., Sollie A., van Suijlekom-Smit L.W., Koning S. (2017). Interventions for cutaneous molluscum contagiosum. Cochrane Database Syst Rev.

